# M. Després

**Published:** 1861-01

**Authors:** 


M. DESPRÉS, Surgeon to the Bicêtre Hospital of Paris, died early in October, in the fiftieth year of his age.

